# Efficacy of daratumumab combination regimen in patients with multiple myeloma: A combined analysis of phase III randomized controlled trials

**DOI:** 10.1002/jha2.46

**Published:** 2020-06-23

**Authors:** Thura W. Htut, Kyaw Z. Thein, Alastair Lawrie, Jane Tighe, Gavin Preston

**Affiliations:** ^1^ Department of Haematology, Aberdeen Royal Infirmary Foresterhill Health Campus Aberdeen UK; ^2^ Department of Investigational Cancer Therapeutics The University of Texas MD Anderson Cancer Center Houston Texas USA

**Keywords:** daratumumab, meta‐analysis, multiple myeloma, phase III randomized controlled trials

## Abstract

The use of the CD38 monoclonal antibody daratumumab in combination with standard myeloma chemotherapy regimens has been studied extensively in recent years. We undertook an updated meta‐analysis of phase III randomized controlled trials (RCT) to determine the efficacy of daratumumab combination regimens. The relative risk for progression was significantly lower in daratumumab‐treated cohorts (HR 0.46, 95% CI 0.38‐0.55) and this was consistent across newly diagnosed and relapsed cases. No statistically significant improvement was identified in newly diagnosed patients with high‐risk cytogenetics and this group remains a therapeutic challenge.

## 1 INTRODUCTION

Multiple myeloma (MM) is a hematological malignancy characterized by monoclonal proliferation of abnormal plasma cells, which accounts for one percent of all cancers worldwide [1]. MM remains incurable and complicated by end‐organ damage, including anemia, hypercalcemia, renal dysfunction, and lytic lesions in the bone [2]. MM ultimately progresses or relapses and remains a therapeutic challenge [3]. Over the past two decades, proteasome inhibitors (PI) and immunomodulatory drugs (IMiDs) have improved survival in patients with MM [4]. Introducing novel agents has recently become the hallmark of a therapeutic paradigm shift in the management of newly diagnosed MM (NDMM) and relapsed or refractory MM (RRMM), in both transplant – eligible and – ineligible patients [5]. Daratumumab is a human immunoglobulin G1 kappa (IgG1κ) monoclonal antibody that can bind to CD38 on the surface of myeloma cells and lead to cell lysis [6]. Studies have shown that daratumumab monotherapy or in combination with PIs, IMiDs, and/ or other anti‐myeloma therapies increased survival in the treatment of MM. We undertook an updated meta‐analysis of phase III randomized controlled trials (RCT) to determine the efficacy of daratumumab combination regimens in patients with NDMM and RRMM.

## 2 METHODS

The systematic review was performed as per the Cochrane Handbook for Systematic Reviews and reported in accordance with the Preferred Reporting Items for Systematic Reviews and Meta‐Analyses (PRISMA) guidelines [7]. We performed systematically a comprehensive literature search using MEDLINE, EMBASE databases and meeting abstracts up to 30^th^ April 2020 using the keywords “multiple myeloma AND daratumumab,” OR “plasma cell disorder AND daratumumab.” The references of all potential studies were also reviewed for any additional relevant studies. We limited the search to “humans” and “randomised controlled trials.” All studies written in English or non‐English languages were obtained. The studies that were eligible to be included in the meta‐analysis had to conform with the following characteristics: phase III RCTs utilizing daratumumab in patients with newly diagnosed/untreated multiple myeloma or relapsed/refractory multiple myeloma.

The primary outcome of our meta‐analysis was progression‐free survival (PFS). The secondary outcome was the overall response rate (ORR), including stringent complete response (sCR), complete response (CR), and MRD negativity (molecular response). We summarized the characteristic features of incorporated studies in Table 1 [8, 9, 10, 11, 12, 13]. Six phase III RCTs (POLLUX, CASTOR, CANDOR, ALCYONE, CASSIOPEIA, and MAIA studies) involving 4025 patients (2094 participants in daratumumab group and 1931 cases in control group) were included in the final analysis. Studies compared daratumumab based combination regimens with antimyeloma regimens without daratumumab as shown in **Table** **1**. Daratumumab was utilized in relapsed and refractory multiple myeloma in the POLLUX, CASTOR, and CANDOR studies, and as first‐line treatment for patients with multiple myeloma in the ALCYONE, CASSIOPEIA, and MAIA studies. The randomization ratio was 1:1 in all studies except 2:1 in the CANDOR trial. Mantel‐Haenszel (MH) method was used to estimate the pooled hazard ratio (HR) for progression‐free survival (PFS), and pooled risk ratio (RR), and risk difference (RD) with 95% confidence interval (CI) for ORR, CR, and sCR and MRD. All statistical analyses were performed using the Review Manager, version 5.3 (Nordic Cochrane Centre; Copenhagen, Denmark). Heterogeneity was assessed with *I* ^2^ and Cochran's Q statistic [14]. A “*P*‐value” of <.05 was considered significant and *I* ^2^ > 50% is considered substantially heterogeneous. An HR < 1.0 or RR < 1.0 was in favor of daratumumab. The risk of bias for each study was evaluated by Cochrane RevMan 5.3 software. Five main salient biases (selection bias, performance bias, detection bias, attrition bias, reporting bias, and others) were categorized and were rated as low, high, or unclear risk [14]. Publication bias was assessed by funnel plots.

**TABLE 1:** Characteristics of the studies included in the meta‐analysis

					Number of Patients			
Study	Author/Year	Study Type	Study Phase	Line of Treatment	Daratumumab	Control	Treatment	
ALCYONE	Mateos/2020	Multicentre, Randomised, open‐label, active‐control	III	Untreated patients who are ineligible for stem cell transplantation	350	356	DVMP	VMP
MAIA	Facon/2019	Randomised, open label, multicentre	III	Newly diagnosed multiple myeloma who were ineligible for autologous stem cell transplantation	368	369	DRDex	RDex
CASSIOPEIA	Moreau/2019	Multicentre, Randomised, open‐label, active‐control	III	Newly diagnosed multiple myeloma who were eligible for autologous stem cell transplantation	543	542	DVTDex	VTDex
POLLUX	Bahlis/2020	Randomised, open‐label, multicentre	III	Relapsed or refractory multiple myeloma	281	276	DRDex	RDex
CASTOR	Spencer/2018	Multicentre, randomised, open‐label, active‐controlled	III	Relapsed or relapsed and refractory multiple myeloma	240	234	DVDex	VDex
CANDOR	Usmani/2019	Randomised, open label,	III	Relapsed or relapsed and refractory multiple myeloma	312	154	KDDex	KDex

Abbreviations: D, daratumumab; V, bortezomib; M, melphalan; P, prednisolone; R, lenalidomide; Dex, dexamethasone; T, thalidomide; K, carfilzomib.

## 3 RESULTS

**FIGURE 1:**
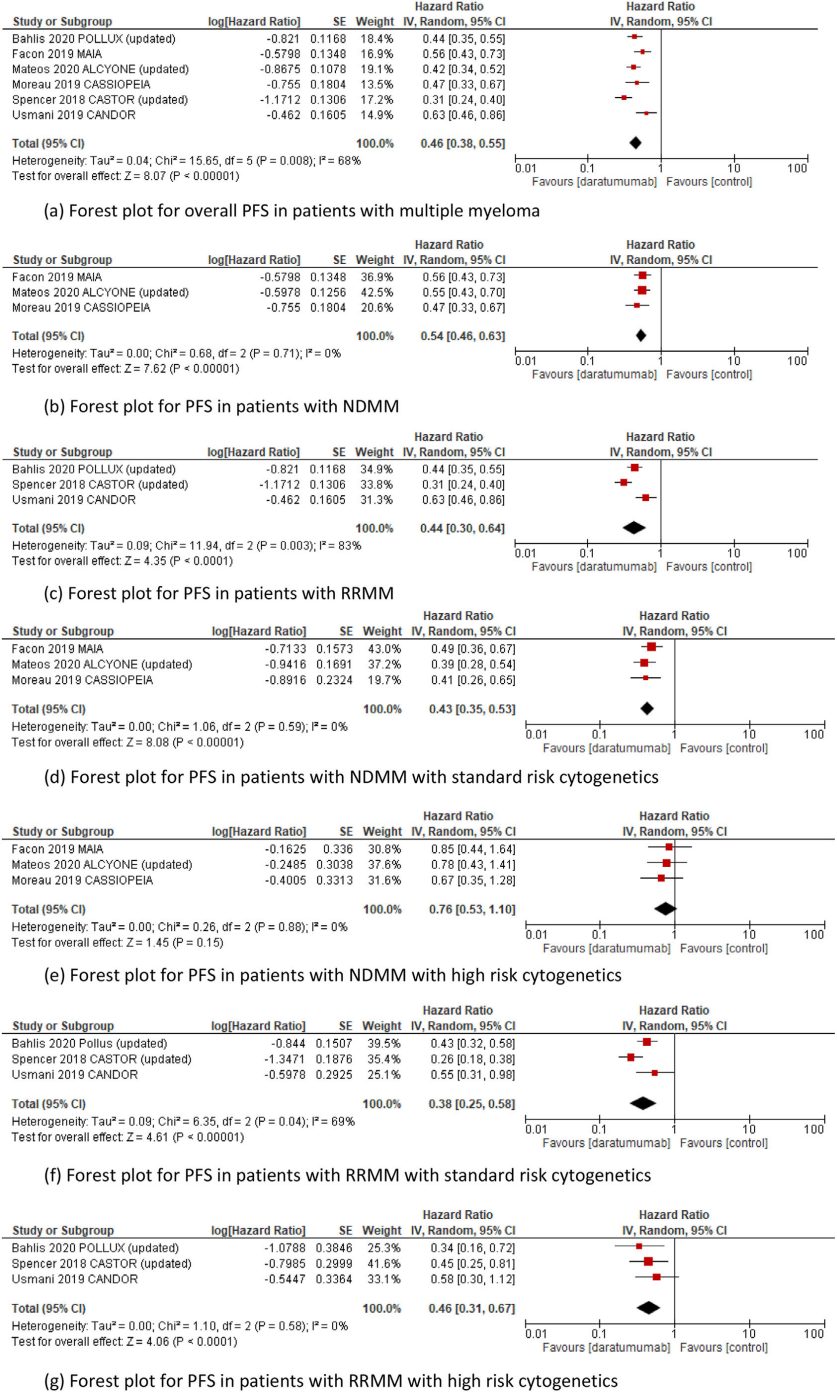
Pooled HR for PFS in patients with multiple myeloma: A, overall; B, NDMM; C, RRMM; D, SRC NDMM; E, HRC NDMM; F, SRC RRMM; G, HRC RRMM receiving daratumumab containing regimen versus control Abbreviations: HR, hazard ratio; PFS, progression free survival; NDMM, newly diagnosed multiple myeloma; RRMM, relapsed and refractory multiple myeloma; SRC, Standard risk cytogenetic; HRC, High risk cytogenetic.

The *I* ^2^ statistic showed some heterogeneity among RCTs and the random‐effects model was applied to provide a more conservative result. The pooled HR for overall PFS was statistically significant at .46 (95% CI: 0.38–0.55; *P* < .00001) Figure 1A. The pooled HR for PFS was calculated for each subset; NDMM in Figure 1B (HR, 0.54; 95% CI: 0.46–0.63; *P* < .00001) and RRMM in Figure 1C (HR, 0.44; 95% CI: 0.30–0.64; *P* < .0001). Although the pooled HR for PFS was significant in standard‐risk cytogenetic NDMM cohort in Figure 1D (HR, 0.43; 95% CI: 0.35–0.53; *P* < .00001), PFS was not statistically significant in high risk cytogenetic NDMM cohort in Figure 1E (HR, 0.76; 95% CI: 0.53–1.10; *P* = .15). A PFS benefit was observed in both standard‐risk cytogenetic and high‐risk cytogenetic cohorts in RRMM with the pooled HR of 0.38 (95% CI: 0.25–0.58; *P* < .00001) and the HR of 0.46 (95% CI: 0.31–0.67; *P* < .0001), respectively in Figure 1F,G. According to an analysis of two trials, which enrolled transplant‐ineligible NDMM patients (ALCYONE and MAIA trials), the pooled HR for PFS was not significant at 0.81 (95% CI: 0.52–1.26; *P* = .35) in patients with NDMM who harbored high‐risk cytogenetics.

The benefit in ORR was observed in both NDMM and RRMM who have received a daratumumab‐containing regimen. In NDMM, ORR was reported in 92.2% in daratumumab arm versus 82.8% in the control arm (RR, 1.13; 95% CI: 1.01–1.26; *P* = .03). In RRMM, ORR was 87% versus 71.3% in the control arm (RR, 1.22; 95% CI: 1.12–1.32; *P* < .00001). In NDMM, the rate of CR and sCR was 17.9% higher in daratumumab combination regimens compared to the control group (RR, 1.71; 95% CI: 1.47–1.99; *P* < .00001), whereas the rate of CR and sCR was 22.5% higher in daratumumab arm in the RRMM subgroup (RR, 2.57; 95% CI: 2.12–3.12; *P* < .00001). Higher MRD 10^−5^ negativity was also observed in both NDMM and RRMM. In NDMM, molecular remission was reported in 38.8% in the daratumumab arm versus 22% in the control arm (RR, 2.49; 95% CI: 1.23‐5.04; *P* = .01). In RRMM, molecular remission was reported in 18.4% of patients in the daratumumab arm versus 3.4% in the control arm and the pooled RR was significant at 5.73 (95% CI: 3.75–8.78; *P* < .00001).

## 4 DISCUSSION

Daratumumab is an anti‐CD38 monoclonal antibody that lyses abnormal plasma cells through direct cytotoxicity to the cell and complement activation [6] as well as an immunomodulatory effect [15]. The antitumor effect of daratumumab can be enhanced by the addition of immunomodulatory drugs such as lenalidomide [6]. Recently, there have been several studies comparing the efficacy and safety of daratumumab combination regimens with non‐daratumumab based anti‐myeloma therapies.

Our meta‐analysis showed that daratumumab combination regimens yielded better PFS than control arms in both NDMM and RRMM. The improvement in PFS was noted across all subgroups except in NDMM with high‐risk cytogenetics. Further analysis revealed that the PFS benefit was not observed in high‐risk cytogenetic NDMM patients regardless of transplant eligibility. Only about 15% of the patients in all three trials were in the high‐risk cytogenetic group, so it remains possible that the HR favoring daratumumab may become statistically significant with greater numbers. There remains a significant need for more novel approaches and therapeutics to address this high‐risk subset.

A higher molecular remission rate of 64% (MRD10^−5^) was reported in the daratumumab group in NDMM in the CASSIOPEIA study compared to 16% in patients with NDMM who received daratumumab combination regimens in the ALCYONE study and 24.1% in the MAIA study. The ALCYONE and MAIA trials enrolled transplant‐ineligible patients and are relatively older populations with the mean age of ∼70 years whereas the CASSIOPEIA study included relatively younger patients with a mean age of 59 years and they were eligible to undergo autologous stem cell transplantation. The differences in patient selection criteria may explain the differences in the rate of response and impact on survival.

Despite the consistency of our findings, some caution should be used in their interpretation. First, the studies used different standard combination regimens, trial design, and methodology, which might confound the analysis. Second, the overall survival data are still immature for a few trials and we used the abstract data for the CANDOR trial. Individual patient data pooled meta‐analysis would provide more detailed and accurate analyses and confirm our findings. Lastly, translating clinical trial data into the real‐world setting is of paramount importance.

## 5 CONCLUSION

Our meta‐analysis showed that daratumumab combination regimens significantly improved PFS, ORR, CR, and sCR, and MRD negativity compared to control arms in patients with NDMM and RRMM. The improvement in PFS was noted across all subgroups except in NDMM with high‐risk cytogenetics. More randomized studies are necessary in the future to explore further novel therapies and the optimal combination of anti‐myeloma therapies to improve survival in patients with NDMM within the high‐risk cytogenetic subset.

## AUTHOR CONTRIBUTIONS

Thura W. Htut and Kyaw Z. Thein contributed to the study conception and design. Material preparation, data collection, and analysis were performed by Thura W. Htut and Kyaw Z. Thein. The first draft of the manuscript was written by Thura W. Htut and Kyaw Z. Thein, and Alastair Lawrie, Jane Tighe, and Gavin Preston commented on the manuscript. All authors read and approved the final manuscript.

## CONFLICTS OF INTEREST AND SOURCE OF FUNDING

Gavin Preston has received honoraria or meeting sponsorship from Abbvie, Janssen‐Cilag, and Takeda, and has attended meetings sponsored by Celgene, Roche, Bristol‐Myers‐Squibb, Novartis, Gilead, Pfizer, and Napp pharmaceuticals. Jane Tighe has received educational support and travel for meetings from Amgen, Celgene, Sanofi Aventis, Takeda, and Janssen over the years. We had no financial support for this project.
